# Socioeconomic Factors and Severity of Coronary Artery Disease in Patients Undergoing Coronary Angiography: A Multicentre Study of Arabian Gulf States

**DOI:** 10.2174/1874192401711010047

**Published:** 2017-04-28

**Authors:** Amin Daoulah, Osama E. Elkhateeb, S. Ali Nasseri, Mushabab Al-Murayeh, Salem Al-kaabi, Amir Lotfi, Mohamed N. Alama, Salem M. Al-Faifi, Mamdouh Haddara, Ciaran M. Dixon, Ibrahim S. Alzahrani, Abdullah A. Alghamdi, Waleed Ahmed, Adnan Fathey, Ejazul Haq, Alawi A Alsheikh-Ali

**Affiliations:** 1 Section of Adult Cardiology, Cardiovascular Department, King Faisal Specialist Hospital & Research Center, Jeddah, Kingdom of Saudi Arabia.; 2 Cardiac Center, King Abdullah Medical City in Holy Capital Makkah, Kingdom of Saudi Arabia.; 3 Politecnico di Torino, Italy Armed Forces Hospital Southern Region, Khamis Mushayt, Kingdom of Saudi Arabia.; 4 Cardiovascular Department, Armed Forces Hospital Southern Region, Khamis Mushayt, Kingdom of Saudi Arabia.; 5 Cardiology Department, Zayed Military Hospital, Abu Dhabi, UAE.; 6 Division of Cardiology, Baystate Medical Center, Tufts University School of Medicine, Springfield, Massachusetts.; 7 Cardiology unit, King Abdul Aziz University Hospital, Jeddah, Kingdom of Saudi Arabia.; 8 Internal Medicine Department, King Faisal Specialist Hospital & Research Center, Jeddah, Kingdom of Saudi Arabia.; 9 Anesthesia Department, King Faisal Specialist Hospital & Research Center, Riyadh, Kingdom of Saudi Arabia.; 10 Emergency Department, King Faisal Specialist Hospital and Research Centre, Riyadh, Kingdom of Saudi Arabia; 11 College of medicine, King Abdul Aziz University Hospital, Jeddah, Kingdom of Saudi Arabia; 12 College of Medicine, Mohammed Bin Rashid University of Medicine and Health Sciences, Dubai, UAE. Institute of Cardiac Sciences, Sheikh Khalifa Medical City, Abu Dhabi, UAE.

**Keywords:** Arabian Gulf, Cross sectional study, Coronary artery disease, Coronary angiography, Cardiac epidemiology, CAD

## Abstract

**Introduction:**

Coronary artery disease (CAD) is a leading cause of death worldwide. The association of socioeconomic status with CAD is supported by numerous epidemiological studies. Whether such factors also impact the number of diseased coronary vessels and its severity is not well established.

**Materials and Methods:**

We conducted a prospective multicentre, multi-ethnic, cross sectional observational study of consecutive patients undergoing coronary angiography (CAG) at 5 hospitals in the Kingdom of Saudi Arabia and the United Arab Emirates. Baseline demographics, socioeconomic, and clinical variables were collected for all patients. Significant CAD was defined as ≥70% luminal stenosis in a major epicardial vessel. Left main disease (LMD) was defined as ≥50% stenosis in the left main coronary artery. Multi-vessel disease (MVD) was defined as having >1 significant CAD.

**Results:**

Of 1,068 patients (age 59 ± 13, female 28%, diabetes 56%, hypertension 60%, history of CAD 43%), 792 (74%) were from urban and remainder (26%) from rural communities. Patients from rural centres were older (61 ± 12 *vs* 58 ± 13), and more likely to have a history of diabetes (63 *vs* 54%), hypertension (74 *vs* 55%), dyslipidaemia (78 *vs* 59%), CAD (50 *vs* 41%) and percutaneous coronary intervention (PCI) (27 *vs* 21%). The two groups differed significantly in terms of income level, employment status and indication for angiography. After adjusting for baseline differences, patients living in a rural area were more likely to have significant CAD (adjusted OR 2.40 [1.47, 3.97]), MVD (adjusted OR 1.76 [1.18, 2.63]) and LMD (adjusted OR 1.71 [1.04, 2.82]). Higher income was also associated with a higher risk for significant CAD (adjusted OR 6.97 [2.30, 21.09]) and MVD (adjusted OR 2.49 [1.11, 5.56]), while unemployment was associated with a higher risk of significant CAD (adjusted OR 2.21, [1.27, 3.85]).

**Conclusion:**

Communal and socioeconomic factors are associated with higher odds of significant CAD and MVD in the group of patients referred for CAG. The underpinnings of these associations (*e.g.* pathophysiologic factors, access to care, and system-wide determinants of quality) require further study.

## INTRODUCTION

CAD is the leading cause of death globally and in the Middle East [1, 2]. Development of CAD has been attributed to known modifiable risk factors [3]. Other factors such as physiological, psychological, emotional, social, and stress both acute and chronic have been studied [4-22]. Acute and chronic psychological stressors increase haemostatic factors and acute phase proteins, leading to CAD [23]. In addition, the interaction between risk factors has consequences [24].

The association between socioeconomic factors and CAD has been supported by numerous epidemiological studies [25-31]. Since the mid-1970s, the rate of major cardiovascular disease (CVD) and death has shifted from high-income to low-income countries [32-36]. In addition, rural rather than urban communities from low-income countries have a higher rate of major CVD and death, whereas no such difference was found in high-income countries [36]. Despite the fact that the risk factor burden was lower in low-income countries compared with high-income countries, the rates of major CVD and death were higher in the low-income countries [36]. This difference may be due to the presence of socioeconomic inequalities in access to treatment for CAD [37]. It is expected by 2020 that >80% of CVD will occur in low-income countries [38].

Therefore, we conducted a study examining the association between urbanization, income level, education and employment status with severity of CAD in the group of patients undergoing CAG for clinical indications in two Arabian Gulf regions.

## MATERIALS AND METHODS

### Study Population

The details regarding the design, methods, and endpoints of this multicentre, observational study came from the Polygamy and Risk of Coronary Artery Disease in Men Undergoing Angiography [39]. In the current study the data were collected prospectively from five hospitals in two Arabian Gulf regions (The Kingdom of Saudi Arabia and The United Arab Emirates), during the period April 1, 2013 to March 30, 2014. Two separate data forms (general and angiographic) were completed by the assigned physician. Both forms were completed before the patients were discharged from hospital. All data forms were reviewed by the respective cardiologist, then sent online to the principal investigator, who also checked the forms before submission for analysis. The study was approved by King Faisal Specialist Hospital and Research Center Institutional Review Board, and an invitation letter was given to all participants who affirmed verbal consent prior to their enrolment. All patients undergoing CAG were recruited; none refused to participate. There were no exclusion criteria.

### Contents of Personal Data Form

Data were collected on demographics (age, ethnic background), physiological status (hypertension, diabetes, dyslipidaemia, BMI), life style (smoking history), past medical history (CAD, PCI, coronary artery bypass graft (CABG), cerebral vascular disease, peripheral arterial disease, congestive heart failure, atrial fibrillation and chronic kidney disease), and urban *vs* rural residence. Rural residence was defined by national statistical offices in Saudi Arabia and was last measured on 17.07.2014, according to the World Bank [40]. It was calculated as the difference between total population and urban population. Socioeconomic data included occupation (unemployed, private sector, government sector, self-employed), highest level of education completed (illiterate, secondary school, undergraduate, masters, PhD), and monthly income (<1300, 1300 to 2600, 2600 to 5300, 5300 to 7900, 7900 to 10600, >10600 US Dollars).

### Contents of Angiographic Data Form

Data collected included; reason for coronary angiography (elective *vs* urgent/emergent), number of vessels involved (severity), and treatment (medical *vs* revascularization).

### Definitions

Significant CAD was defined as ≥70% luminal stenosis in a major epicardial vessel. LMD was defined as ≥50% stenosis in the left main coronary artery. MVD was defined as having >1 coronary artery with significant disease. Non-ST-segment-elevation acute coronary syndrome (NSTEACS) includes non-ST-segment-elevation myocardial infarction (NSTEMI) and unstable angina.

### Statistical Analysis

Continuous variables are summarized using means and standard deviations, and compared with the Student’s t-test. Categorical variables are summarized using percentages and compared with the Chi-square test. The associations between urbanization and other socioeconomic factors such as income, employment, education level and severity of CAD, MVD and LMD were assessed using logistic regression models and quantified with odds ratios. Adjusted regression models included the following explanatory variables: age, gender, baseline risk factors, past history of CAD/PCI or CABG, ethnicity, marital status, and indication for angiography (elective *vs* urgent/emergent). All statistical tests were two-sided and significance was set at the conventional 0.05. No adjustments for multiple comparisons were made.

## RESULTS

### Descriptive

Overall characteristics of patients and coronary angiogram findings are shown in (Table **1**). A detailed description can be found in Polygamy and Risk of Coronary Artery Disease in Men Undergoing Angiography [39].

Table (**1**) shows the patients characteristics stratified by urbanization and other socioeconomic factors. We enrolled 1,068 patients, 792 (74%) were from urban areas, and 276 (26%) from rural areas. Patients from rural areas were older (61 ± 12 *vs* 58 ± 13), and more likely to have a history of diabetes (63 *vs* 54%), hypertension (74 *vs* 55%), dyslipidaemia (78 *vs* 59%), CAD (50 *vs* 41%), and PCI (27 *vs* 21%). The two groups did not differ significantly in terms of smoking, cerebral vascular disease, chronic kidney disease, peripheral arterial disease, atrial fibrillation, or history of depression. The indications for CAG differed significantly between the two groups. Of the rural population, 59% had CAG for non-urgent/emergent indication as opposed to 44% of the urban population, p <0.0001. Both groups did not differ when compared according to the number of vessel involved during CAG; overall, 67% of the urban population and 72% of the rural population had CAD. The presence of single or multi-vessel CAD in both populations not differ significantly. Presence of LM disease was higher in rural population compared with the urban population (16 *vs* 10%), p=0.0065. Types of intervention differed significantly between the two groups. Of the urban population, 38% received medical therapy compared with 33% of the rural population. However, 48% of the rural population had PCI as compared with 43% of the urban population, p<0.0001.

Multivariate logistic analysis was used to adjust data for baseline differences and characterize the odds of significant CAD, MVD, and LMD as it relates with the socio-economic factors under study. Tables (**2**-**4**) summarize the results of this regression analysis for significant CAD, MVD and LMD, respectively. Patients living in a rural area were more likely to have significant CAD (OR 2.40 [1.47, 3.97]), MVD (OR 1.76 [1.18, 2.63]) and LMD (OR 1.71 [1.04, 2.82]). Higher income was also associated with higher odds for significant (CAD [OR 6.97 [2.30, 21.09]) and MVD (OR 2.49 [1.11, 5.56]), while unemployment was associated with a higher risk of significant CAD (OR 2.21 [1.27, 3.85]). There was no clear association between significant CAD and level of education.

## DISCUSSION

The association between socioeconomic factors and CAD has been demonstrated by many epidemiological studies of both developed and developing countries [25-31]. O'Connor *et al.* examined the rural-urban differences in the prevalence of CAD in >214,000 respondents using data from the US Centers for Disease Control and Prevention's (CDC's) 2008 Behavioral Risk Factor Surveillance System. They found a higher prevalence of CAD in rural populations [41]. In another study, rural rather than urban communities from low-income countries have a higher rate of major CVD and death whereas no such difference was found between rural and urban communities in high-income countries [36]. Our study reported a higher risk of significant CAD (OR 2.40), MVD (OR 1.76) and LMD (OR 1.71) in those living in a rural area compared with those living in urban area. This could be attributed to the delay in seeking health care, even when experiencing symptoms of a suspected heart attack [42] or due to poor access to medical facilities and regular screening programs in these areas [43-45].

The rate of CVD during the period from the 1930s to the 1950s was low in low-income countries and high in high-income countries [32, 33]. Since the mid-1970s the rate of CVD has declined in several high-income countries, owing to reductions in risk factors and better management of CVD [34]. In contrast, the rate of CVD increased in some low-income countries [46, 47] with 80% of the global burden estimated to occur in these countries [38]. This may be due to the presence of socioeconomic inequalities in access to treatment for CAD. Patients with low socioeconomic status are less frequently referred for CAG and subsequent management (*i.e.* revascularization and secondary prevention) [37]. In addition, there is a delay in hospital presentation after onset of symptoms of acute myocardial infarction [48]. However, our study demonstrated a link between higher income and higher odds for significant CAD (OR 6.97) and MVD (OR 2.49). We speculate that this finding could be explained by poor lifestyle (physical inactivity and a sedentary lifestyle, unhealthy dietary intake, tobacco use both smoking and non-smoking, and second hand tobacco smoke) [49-51] and the higher stressful events related to work and daily activities that individuals with higher income living in the Gulf region exhibit. Previous studies among men linking stress-related health risks with substantial losses in income and wealth [52, 53] help support our speculation. Based on our study, we believe further investigation is required to understand the effect of income and wealth on CAD and to evaluate the underlying mechanisms that lead to its effects.

Our study demonstrated a higher risk of CAD (OR 2.21) associated with unemployment, which is in agreement with previous studies [54-57]. Socioeconomic factors also seem to be implicated in the relationship between education and cardiac health [58, 59]. Recently, the US National Bureau of Economic Research stated that each additional 4 years of education lowered all-cause mortality by almost 1.8% and reduced the risk of heart disease by 2.2% [60]. However, our current study failed to show the association between education level and risk of CAD, and this could be explained by similar distribution of education levels in the rural and urban population, and the large number of illiterate people in our study population.

It has been suggested that socioeconomic factors exert their impact on the heart through affecting the central nervous system, increasing output from the sympathetic nervous system and the hypothalamic-pituitary-adrenal (HPA) axis. Chronic stimulation from these outputs can induce a wide variety of pathophysiologic responses, including inflammation and platelet activation [11, 61-63]. They may also affect health related behaviours such as smoking, diet, alcohol consumption and physical activity, and affect access to health care which might be affected by social support [64]. Further study is required to understand the underlying mechanisms that lead to the association of high income in the gulf region with higher CAD risk. This might suggest that the well-accepted socioeconomic-CAD gradient might not be applicable to all regions of the world. Such differences in risk factors between different ethnicities are not uncommon and have been seen for instance in the South Asian and African population in the UK [65-67]. Another possible explanation of such differences might be attributed to the way socioeconomic status was taken into account. It is important for the health care providers when discussing traditional risk factors, to incorporate discussion of different aspect of socioeconomic factor management as part of the overall cardiovascular health [11, 30].

### Study Strengths

This study is the first to look at the association between socioeconomic factors and severity of CAD in the group of patients referred for CAG for clinical indications in two gulf regions.

### Study Limitations

First, it is surprising that almost half the cohort were illiterate and approximately 90% had a low monthly income: this is likely due to a small sample size and referral bias. One possible explanation is the nature of the enrolled hospitals, which tend to receive referrals from poorer areas. Second, the time interval from the socioeconomic factors to the cardiac catheterization was not recorded; this interval may have influenced the findings. Third, our study population was selected to undergo CAG if clinically indicated, and as such, cannot be generalized to all the population in the Gulf Region. Fourth, we relied on self-reporting history of depression, without using a formal objective screening tool, thus the incidence of depression in our cohort may not be accurate. Fifth, we did not look at unmeasured confounding variables such as dietary habits, physical activity, inflammatory markers, and other unconsidered variables. Chronic stress was found to reduce the levels of leptin receptors [68] and increase levels of peripheral neuropeptide Y [69] which might change dietary habit and lead to consumption of high fat diet and increase risk of CAD. Several studies also indicate that the stress impedes individual efforts to be more physically active, increasing risk of CAD [70]. Stress has been associated with higher levels of inflammatory markers such as C-reactive protein (CRP), TNF-α [71-73] and pro-inflammatory cytokines [72], which contribute to increased risk of CAD. Six, we did not obtain information specifically regarding retirement or those receiving industrial injury benefit in defining employment status.

## CONCLUSION

Communal and socioeconomic factors are associated with severity of CAD and MVD in the group of patients referred for CAG for clinical indications in two gulf regions. The well-accepted socioeconomic-CAD gradient might not be applicable to all regions of the world. We suggest that the interpretation of socioeconomic status should take in account the differences in risk factors between different ethnicities and the difference of cultural life style in individuals from the same socioeconomic status. The underpinnings of these associations (*e.g.* pathophysiologic factors, access to care, and system-wide determinants of quality) require further study.

## Figures and Tables

**Table 1:** Overall patient characteristic Stratified by Urbanization and other socioeconomic factors.

	**All Patients** **(n=1,068)**	**Urban** **(n=792)**	**Rural** **(n=276)**	**P**
**Age**	59 ± 13	58 ± 13	61 ± 12	0.0107
**Male (%)**	73%	71%	77%	0.0667
**BMI**	28 ± 6	28 ± 6	28 ± 6	0.259
**Diabetes Mellitus (%)**	56%	54%	63%	0.0131
**Hypertension (%)**	60%	55%	74%	<0.0001
**Smoking (%)**	43%	42%	47%	0.406
**Dyslipidaemia (%)**	64%	59%	78%	<0.0001
**Past History (%)**				
**Coronary Artery Disease**	43%	41%	50%	0.0183
**PCI**	23%	21%	27%	0.0305
**CABG**	6%	6%	7%	0.5375
**Atrial Fibrillation**	6%	7%	4%	0.1799
**CHF**	14%	13%	17%	0.0675
**CVA**	5%	5%	4%	0.6356
**CKD**	16%	16%	15%	0.7141
**Depression**	9%	10%	8%	0.3565
**PAD**	4%	4%	3%	0.4203
**Ethnicity (%)**				0.0587
**Gulf National**	89%	88%	91%	
**Other Arab**	5%	6%	3%	
**Non-Arab**	6%	6%	7%	
**Monthly Income Category (%)**				<0.0001
**A) < *$*1300**	58%	62%	48%	
**B) *$*1300-2600**	24%	23%	28%	
**C) *$*2600-5300**	11%	10%	13%	
**D) > *$*5300**	7%	5%	11%	
**Job Category (%)**				<0.0001
A) **Unemployed**	39%	42%	30%	
B) **Private sector**	13%	15%	8%	
C) **Government**	35%	26%	60%	
D) **Self-employed**	13%	17%	2%	
**Education Level (%)**				0.6976
A) **Illiterate**	49%	50%	46%	
B) **Secondary school**	34%	33%	37%	
C) **Undergraduate**	13%	13%	14%	
D) **Master**	3%	3%	2%	
E) **PhD**	1%	1%	1%	
**Indication for CAG (%)**				<0.0001
**Elective**	48%	44%	59%	
**NSTEACS**	46%	49%	39%	
**STEMI**	6%	7%	2%	
**Findings on CAG (%)**				0.144
**No CAD**	32%	33%	28%	
**Single Vessel**	20%	21%	19%	
**Double Vessel**	25%	24%	26%	
**Triple Vessel**	23%	22%	27%	
**Multi-vessel**	48%	46%	53%	0.0345
**Left Main**	12%	10%	16%	0.0065
**Intervention**				<0.0001
**Medical Therapy Only**	37%	38%	33%	
**PCI**	45%	43%	48%	
**CABG**	18%	18%	19%	

**Table 2:** Multivariate logistic regressions [adjusting for age, gender, baseline risk factors, past history of CAD/PCI or CABG, ethnicity, marital status, and indication for CAG (elective *vs* urgent/emergent)] calculating odd of any coronary artery disease.

	**Adjusted**	**95% Confidence**	**P**
	**Odds Ratio**	**Limits**	
**Rural *vs* Urban**	2.4	[1.47, 3.97]	0.0005
**Income > $5300 *vs* < $1300**	6.97	[2.3, 21.09]	0.0007
**Job**			0.0029
**Self-employed *vs* Unemployed**	0.24	[0.10, 0.55]	
**Government *vs* Unemployed**	0.49	[0.27, 0.91]	
**Private sector *vs* Unemployed**	0.87	[0.37, 2.04]	
**Education**			0.1141
**PhD *vs* Illiterate**	0.52	[0.06, 4.31]	
**Master *vs* Illiterate**	0.21	[0.06, 0.75]	
**Undergraduate *vs* Illiterate**	0.94	[0.46, 1.90]	
**Secondary school *vs* Illiterate**	0.68	[0.40, 1.14]	

**Table 3:** Multivariate logistic regressions [adjusting for age, gender, baseline risk factors, past history of CAD/PCI or CABG, ethnicity, marital status, and indication for CAG (elective *vs* urgent/emergent)] calculating odds of multi-vessel disease.

	**Adjusted**	**95% Confidence**	**p**
	**Odds Ratio**	**Limits**	
**Rural *vs* Urban**	1.76	[1.18, 2.63]	0.0058
**Income > $5300 *vs* < $1300**	2.49	[1.11, 5.56]	0.0015
**Job**			0.5159
**Self-employed *vs* Unemployed**	0.67	[0.39, 1.16]	
**Government *vs* Unemployed**	0.79	[0.49, 1.26]	
**Private sector *vs* Unemployed**	0.87	[0.48, 1.59]	
**Education**			0.086
**PhD *vs* Illiterate**	1.79	[0.32, 10.05]	
**Master *vs* Illiterate**	0.36	[0.13, 1.03]	
**Undergraduate *vs* Illiterate**	1.13	[0.65, 1.98]	
**Secondary school *vs* Illiterate**	0.72	[0.50, 1.05]	

**Table 4:** Multivariate logistic regressions [adjusting for age, gender, baseline risk factors, past history of CAD/PCI or CABG, ethnicity, marital status, and indication for CAG (elective *vs* urgent/emergent)] calculating odds of left main disease.

	**Adjusted**	**95% Confidence**	**p**
	**Odds Ratio**	**Limits**	
**Rural *vs* Urban**	1.71	[1.04, 2.82]	0.0355
**Income > $5300 *vs* < $1300**	0.57	[0.20, 1.61]	0.4311
**Job**			0.3886
**Self-employed *vs* Unemployed**	1.14	[0.51, 2.53]	
**Government *vs* Unemployed**	1.02	[0.55, 1.90]	
**Private sector *vs* Unemployed**	1.95	[0.86, 4.42]	
**Education**			0.6484
**PhD *vs* Illiterate**	1.63	[0.25, 10.65]	
**Master *vs* Illiterate**	0.28	[0.04, 2.25]	
**Undergraduate *vs* Illiterate**	0.91	[0.43, 1.32]	
**Secondary school *vs* Illiterate**	0.79	[0.47, 1.32]	

## LIST OF ABBREVIATIONS

BMI: = Body Mass Index
CAD: = Coronary Artery Disease
PCI: = Percutaneous Coronary Intervention
CABG: = Coronary artery bypass grafting
CHF: = Congestive Heart failure
CVA: = Cerebrovascular accident
CKD: = Chronic Kidney Disease
PAD: = Peripheral Arterial disease
$: = USA Dollars
PhD: = A Doctor of Philosophy
STEMI: = ST Segment Elevation Myocardial Infarction
NSTEACS: = Non–ST-Segment Elevation Acute Coronary Syndromes
CAG: = Coronary Angiography

## References

[1] Heron M. (2015). Deaths: Leading Causes for 2012.. Natl. Vital Stat. Rep..

[2] Mokdad A.H., Jaber S., Aziz M.I., AlBuhairan F., AlGhaithi A., AlHamad N.M., Al-Hooti S.N., Al-Jasari A., AlMazroa M.A., AlQasmi A.M., Alsowaidi S., Asad M., Atkinson C., Badawi A., Bakfalouni T., Barkia A., Biryukov S., El Bcheraoui C., Daoud F., Forouzanfar M.H., Gonzalez-Medina D., Hamadeh R.R., Hsairi M., Hussein S.S., Karam N., Khalifa S.E., Khoja T.A., Lami F., Leach-Kemon K., Memish Z.A., Mokdad A.A., Naghavi M., Nasher J., Qasem M.B., Shuaib M., Al Thani A.A., Al Thani M.H., Zamakhshary M., Lopez A.D., Murray C.J. (2014). The state of health in the Arab world, 19902010: An analysis of the burden of diseases, injuries, and risk factors.. Lancet.

[3] Yusuf S., Hawken S., Ounpuu S., Dans T., Avezum A., Lanas F., McQueen M., Budaj A., Pais P., Varigos J., Lisheng L., INTERHEART Study Investigators (2004). Effect of potentially modifiable risk factors associated with myocardial infarction in 52 countries (the INTERHEART study): Case-control study.. Lancet.

[4] Hemingway H., Marmot M. (1999). Evidence based cardiology: Psychosocial factors in the aetiology and prognosis of coronary heart disease. Systematic review of prospective cohort studies.. BMJ.

[5] Rozanski A., Blumenthal J.A., Kaplan J. (1999). Impact of psychological factors on the pathogenesis of cardiovascular disease and implications for therapy.. Circulation.

[6] Krantz D.S., Sheps D.S., Carney R.M., Natelson B.H. (2000). Effects of mental stress in patients with coronary artery disease: Evidence and clinical implications.. JAMA.

[7] Krantz D.S., McCeney M.K. (2002). Effects of psychological and social factors on organic disease: A critical assessment of research on coronary heart disease.. Annu. Rev. Psychol..

[8] Lee S., Colditz G.A., Berkman L.F., Kawachi I. (2003). Caregiving and risk of coronary heart disease in U.S. women: A prospective study.. Am. J. Prev. Med..

[9] Strike P.C., Steptoe A. (2004). Psychosocial factors in the development of coronary artery disease.. Prog. Cardiovasc. Dis..

[10] Kuper H., Marmot M., Hemingway H. (2002). Systematic review of prospective cohort studies of psychosocial factors in the etiology and prognosis of coronary heart disease.. Semin. Vasc. Med..

[11] Kubzansky L.D., Davidson K.W., Rozanski A. (2005). The clinical impact of negative psychological states: Expanding the spectrum of risk for coronary artery disease.. Psychosom. Med..

[12] Rozanski A., Blumenthal J.A., Davidson K.W., Saab P.G., Kubzansky L. (2005). The epidemiology, pathophysiology, and management of psychosocial risk factors in cardiac practice: The emerging field of behavioral cardiology.. J. Am. Coll. Cardiol..

[13] Holmes S.D., Krantz D.S., Rogers H., Gottdiener J., Contrada R.J. (2006). Mental stress and coronary artery disease: A multidisciplinary guide.. Prog. Cardiovasc. Dis..

[14] Bhattacharyya M.R., Steptoe A. (2007). Emotional triggers of acute coronary syndromes: Strength of evidence, biological processes, and clinical implications.. Prog. Cardiovasc. Dis..

[15] Davidson K.W. (2008). Emotional predictors and behavioral triggers of acute coronary syndrome.. Cleve. Clin. J. Med..

[16] Steptoe A., Kivimäki M. (2012). Stress and cardiovascular disease.. Nat. Rev. Cardiol..

[17] Orth-Gomér K., Wamala S.P., Horsten M., Schenck-Gustafsson K., Schneiderman N., Mittleman M.A. (2000). Marital stress worsens prognosis in women with coronary heart disease: The Stockholm Female Coronary Risk Study.. JAMA.

[18] Dimsdale J.E. (2008). Psychological stress and cardiovascular disease.. J. Am. Coll. Cardiol..

[19] Phillips J.E., Klein W.M. (2010). Socioeconomic Status and Coronary Heart Disease Risk: The Role of Social Cognitive Factors.. Soc. Personal. Psychol. Compass.

[20] Bajekal M., Scholes S., Love H., Hawkins N., OFlaherty M., Raine R., Capewell S. (2012). Analysing recent socioeconomic trends in coronary heart disease mortality in England, 20002007: A population modelling study.. PLoS Med..

[21] Kivimäki M., Nyberg S.T., Batty G.D., Fransson E.I., Heikkilä K., Alfredsson L., Bjorner J.B., Borritz M., Burr H., Casini A., Clays E., De Bacquer D., Dragano N., Ferrie J.E., Geuskens G.A., Goldberg M., Hamer M., Hooftman W.E., Houtman I.L., Joensuu M., Jokela M., Kittel F., Knutsson A., Koskenvuo M., Koskinen A., Kouvonen A., Kumari M., Madsen I.E., Marmot M.G., Nielsen M.L., Nordin M., Oksanen T., Pentti J., Rugulies R., Salo P., Siegrist J., Singh-Manoux A., Suominen S.B., Väänänen A., Vahtera J., Virtanen M., Westerholm P.J., Westerlund H., Zins M., Steptoe A., Theorell T., IPD-Work Consortium (2012). Job strain as a risk factor for coronary heart disease: A collaborative meta-analysis of individual participant data.. Lancet.

[22] Franks P., Winters P.C., Tancredi D.J., Fiscella K.A. (2011). Do changes in traditional coronary heart disease risk factors over time explain the association between socio-economic status and coronary heart disease?. BMC Cardiovasc. Disord..

[23] Ho R.C., Neo L.F., Chua A.N., Cheak A.A., Mak A. (2010). Research on psychoneuroimmunology: Does stress influence immunity and cause coronary artery disease?. Ann. Acad. Med. Singapore.

[24] Kivimäki M., Nyberg S.T., Fransson E.I., Heikkilä K., Alfredsson L., Casini A., Clays E., De Bacquer D., Dragano N., Ferrie J.E., Goldberg M., Hamer M., Jokela M., Karasek R., Kittel F., Knutsson A., Koskenvuo M., Nordin M., Oksanen T., Pentti J., Rugulies R., Salo P., Siegrist J., Suominen S.B., Theorell T., Vahtera J., Virtanen M., Westerholm P.J., Westerlund H., Zins M., Steptoe A., Singh-Manoux A., Batty G.D., IPD-Work Consortium (2013). Associations of job strain and lifestyle risk factors with risk of coronary artery disease: A meta-analysis of individual participant data.. CMAJ.

[25] Mayer O., Šimon J., Heidrich J., Cokkinos D.V., De Bacquer D., EUROASPIRE II Study Group (2004). Educational level and risk profile of cardiac patients in the EUROASPIRE II substudy.. J. Epidemiol. Community Health.

[26] Tubek S., Stepkowski M., Szczurowska A., Storek M., Rzasa A., Matyjaszczyk M., Pociupany R., Wilkins A., Banasiak W., Ponikowski P., Jankowska E.A. (2015). Sexual dimorphism in socioeconomic differences regarding the risk factors, symptomatology and management of patients with stable coronary artery disease in Poland.. Cardiol. J..

[27] Loucks E.B., Lynch J.W., Pilote L., Fuhrer R., Almeida N.D., Richard H., Agha G., Murabito J.M., Benjamin E.J. (2009). Life-course socioeconomic position and incidence of coronary heart disease: The Framingham Offspring Study.. Am. J. Epidemiol..

[28] Ranjit N., Diez-Roux A.V., Shea S., Cushman M., Seeman T., Jackson S.A., Ni H. (2007). Psychosocial factors and inflammation in the multi-ethnic study of atherosclerosis.. Arch. Intern. Med..

[29] Hamer M., Molloy G.J., Stamatakis E. (2008). Psychological distress as a risk factor for cardiovascular events: Pathophysiological and behavioral mechanisms.. J. Am. Coll. Cardiol..

[30] Ferrie J.E., Martikainen P., Shipley M.J., Marmot M.G. (2005). Self-reported economic difficulties and coronary events in men: Evidence from the Whitehall II study.. Int. J. Epidemiol..

[31] Kuper H., Marmot M. (2003). Job strain, job demands, decision latitude, and risk of coronary heart disease within the Whitehall II study.. J. Epidemiol. Community Health.

[32] Walker A.R., Walker B.F., Segal I. (2004). Some puzzling situations in the onset, occurrence and future of coronary heart disease in developed and developing populations, particularly such in sub-Saharan Africa.. J. R. Soc. Promot. Health.

[33] Marmot M., Marmot M., Elliot P. (1992). Coronary heart disease: rise and fall of a modern epidemic.. Coronary heart disease epidemiology: from aetiology to public health..

[34] OFlaherty M., Buchan I., Capewell S. (2013). Contributions of treatment and lifestyle to declining CVD mortality: Why have CVD mortality rates declined so much since the 1960s?. Heart.

[35] Marmot M.G., Adelstein A.M., Robinson N., Rose G.A. (1978). Changing social-class distribution of heart disease.. BMJ.

[36] Yusuf S., Rangarajan S., Teo K., Islam S., Li W., Liu L., Bo J., Lou Q., Lu F., Liu T., Yu L., Zhang S., Mony P., Swaminathan S., Mohan V., Gupta R., Kumar R., Vijayakumar K., Lear S., Anand S., Wielgosz A., Diaz R., Avezum A., Lopez-Jaramillo P., Lanas F., Yusoff K., Ismail N., Iqbal R., Rahman O., Rosengren A., Yusufali A., Kelishadi R., Kruger A., Puoane T., Szuba A., Chifamba J., Oguz A., McQueen M., McKee M., Dagenais G., PURE Investigators (2014). Cardiovascular risk and events in 17 low-, middle-, and high-income countries.. N. Engl. J. Med..

[37] Schröder S.L., Richter M., Schröder J., Frantz S., Fink A. (2016). Socioeconomic inequalities in access to treatment for coronary heart disease: A systematic review.. Int. J. Cardiol..

[38] Murray C.J., Lopez A.D. (1997). Mortality by cause for eight regions of the world: Global Burden of Disease Study.. Lancet.

[39] Daoulah A, Lotfi A, Al-Murayeh M (2017). Polygamy and Risk of Coronary Artery Disease in Men Undergoing Angiography: An Observational
Study. Int. J. Vasc. Med..

[40] Abdul Salam A., Elsegaey I., Khraif R. (2014). Population distribution and household conditions in Saudi Arabia: Reflections from the 2010 Census.. Springerplus.

[41] O'Connor A., Wellenius G. (2012). Rural-urban disparities inthe prevalence of diabetes and coronary heart disease.. Public Health.

[42] Finn JC, Bett JH, Shilton TR, Cunningham C, Thompson PL, Finn J.C., Bett J.H., Shilton T.R., Cunningham C., Thompson P.L., National Heart Foundation of Australia Chest Pain Every Minute Counts Working Group (2007). Patient delay in responding to symptoms of possible heart attack: Can we reduce time to care?. Med. J. Aust..

[43] Weeks W.B., Wallace A.E., Wang S., Lee A., Kazis L.E. (2006). Rural-urban disparities in health-related quality of life within disease categories of Veterans.. J. Rural Health.

[44] Silver M.P., Babitz M.E., Magill M.K. (1997). Ambulatory care sensitive hospitalization rates in the aged Medicare population in Utah, 1990 to 1994: A rural-urban comparison.. J. Rural Health.

[45] Chan L., Hart L.G., Goodman D.C. (2006). Geographic access to health care for rural Medicare beneficiaries.. J. Rural Health.

[46] Stringhini S., Viswanathan B., Gédéon J., Paccaud F., Bovet P. (2013). The social transition of risk factors for cardiovascular disease in the African region: Evidence from three cross-sectional surveys in the Seychelles.. Int. J. Cardiol..

[47] Krishnamurthi R.V., Feigin V.L., Forouzanfar M.H., Mensah G.A., Connor M., Bennett D.A., Moran A.E., Sacco R.L., Anderson L.M., Truelsen T., ODonnell M., Venketasubramanian N., Barker-Collo S., Lawes C.M., Wang W., Shinohara Y., Witt E., Ezzati M., Naghavi M., Murray C., Global Burden of Diseases, Injuries, Risk Factors Study 2010 (GBD 2010), GBD Stroke Experts Group (2013). Global and regional burden of first-ever ischaemic and haemorrhagic stroke during 19902010: Findings from the Global Burden of Disease Study 2010.. Lancet Glob. Health.

[48] Sheifer S.E., Rathore S.S., Gersh B.J., Weinfurt K.P., Oetgen W.J., Breall J.A., Schulman K.A. (2000). Time to presentation with acute myocardial infarction in the elderly: Associations with race, sex, and socioeconomic characteristics.. Circulation.

[49] Cheng X., Li W., Guo J., Wang Y., Gu H., Teo K., Liu L., Yusuf S., INTERHEART China study Investigators (2014). Physical activity levels, sport activities, and risk of acute myocardial infarction: Results of the INTERHEART study in China.. Angiology.

[50] Iqbal R., Anand S., Ounpuu S., Islam S., Zhang X., Rangarajan S., Chifamba J., Al-Hinai A., Keltai M., Yusuf S., INTERHEART Study Investigators (2008). Dietary patterns and the risk of acute myocardial infarction in 52 countries: Results of the INTERHEART study.. Circulation.

[51] Teo K.K., Ounpuu S., Hawken S., Pandey M.R., Valentin V., Hunt D., Diaz R., Rashed W., Freeman R., Jiang L., Zhang X., Yusuf S., INTERHEART Study Investigators (2006). Tobacco use and risk of myocardial infarction in 52 countries in the INTERHEART study: A case-control study.. Lancet.

[52] Cohen S., Janicki-Deverts D. (2012). Who's Stressed? Distributions of Psychological Stress in the United States in Probability Samples from 1983, 2006, and 2009.. J. Appl. Soc. Psychol..

[53] Martikainen P., Laaksonen M., Lahelma E., Rahkonen O. (2010). The associations of household wealth and income with self-rated healthA study on economic advantage in middle-aged Finnish men and women.. Soc. Sci. Med..

[54] Vågerö D, Garcy AM (2016). Does unemployment cause long-term mortality? Selection and causation after the 1992-96 deep Swedish recession.. Eur. J. Public Health..

[55] Lundin A., Falkstedt D., Lundberg I., Hemmingsson T. (2014). Unemployment and coronary heart disease among middle-aged men in Sweden: 39 243 men followed for 8 years.. Occup. Environ. Med..

[56] (2012). Job losses linked to myocardial infarction in US cohort.. BMJ.

[57] Dupre M.E., George L.K., Liu G., Peterson E.D. (2012). The cumulative effect of unemployment on risks for acute myocardial infarction.. Arch. Intern. Med..

[58] World Health Organization The determinants of health.. Available from: http://www.who.int/hia/ evidence/doh/en/.

[59] Beauchamp A., Peeters A., Wolfe R., Turrell G., Harriss L.R., Giles G.G., English D.R., McNeil J., Magliano D., Harrap S., Liew D., Hunt D., Tonkin A. (2010). Inequalities in cardiovascular disease mortality: The role of behavioural, physiological and social risk factors.. J. Epidemiol. Community Health.

[60] (2014). National Bureau of Economic Research The effects of education on health. 2. Available from: http://www.nber.org/digest/mar07/w12352.html.

[61] Hansson G.K. (2005). Inflammation, atherosclerosis, and coronary artery disease.. N. Engl. J. Med..

[62] Brydon L., Magid K., Steptoe A. (2006). Platelets, coronary heart disease, and stress.. Brain Behav. Immun..

[63] Thrall G., Lane D., Carroll D., Lip G.Y. (2007). A systematic review of the effects of acute psychological stress and physical activity on haemorheology, coagulation, fibrinolysis and platelet reactivity: Implications for the pathogenesis of acute coronary syndromes.. Thromb. Res..

[64] Mozaffarian D., Benjamin E.J., Go A.S. (2015). Heart disease and stroke statistics-2015 update: A report from the American Heart Association.. Circulation.

[65] Kuppuswamy V.C., Gupta S. (2005). Excess coronary heart disease in South Asians in the United Kingdom.. BMJ.

[66] Lip G.Y., Barnett A.H., Bradbury A., Cappuccio F.P., Gill P.S., Hughes E., Imray C., Jolly K., Patel K. (2007). Ethnicity and cardiovascular disease prevention in the United Kingdom: A practical approach to management.. J. Hum. Hypertens..

[67] Whincup P.H., Gilg J.A., Papacosta O., Seymour C., Miller G.J., Alberti K.G., Cook D.G. (2002). Early evidence of ethnic differences in cardiovascular risk: Cross sectional comparison of British South Asian and white children.. BMJ.

[68] Yang J.L., Liu X., Jiang H., Pan F., Ho C.S., Ho R.C. (2016). The Effects of High-fat-diet Combined with Chronic Unpredictable Mild Stress on Depression-like Behavior and Leptin/LepRb in Male Rats.. Sci. Rep..

[69] Lu Y., Ho R.C. (2016). An association between neuropeptide Y levels and leukocyte subsets in stress-exacerbated asthmatic mice.. Neuropeptides.

[70] Stults-Kolehmainen M.A., Sinha R. (2014). The effects of stress on physical activity and exercise.. Sports Med..

[70] Gouin J.P., Glaser R., Malarkey W.B., Beversdorf D., Kiecolt-Glaser J. (2012). Chronic stress, daily stressors, and circulating inflammatory markers.. Health Psychol..

[72] Steptoe A., Hamer M., Chida Y. (2007). The effects of acute psychological stress on circulating inflammatory factors in humans: A review and meta-analysis.. Brain Behav. Immun..

[73] McDade T.W., Hawkley L.C., Cacioppo J.T. (2006). Psychosocial and behavioral predictors of inflammation in middle-aged and older adults: The Chicago health, aging, and social relations study.. Psychosom. Med..

